# Visible Spectrophotometric Estimation of Diacerein in Bulk and Pharmaceutical Dosage Forms

**DOI:** 10.4103/0975-1483.71631

**Published:** 2010

**Authors:** R Sivakumar, PK Nallasivan, KC Saranya, Solomon WD Sam, T Akelesh, R Venkatnarayanan

**Affiliations:** *Department of Pharmaceutical Analysis, RVS College of Pharmaceutical Sciences, Sulur, Coimbatore - 641 402, Tamilnadu, India*

**Keywords:** Diacerein, FC reagent, KMnO_4_, spectrophotometry

## Abstract

Two simple, sensitive, accurate, rapid, and economical spectrophotometric methods have been developed for the estimation of diacerein in Pharmaceutical dosage forms. Method A is based on the reaction of diacerein with Folin-Ciocalteu reagent, in the presence of 0.5 N sodium hydroxide solution, giving a pink-colored chromogen, which shows maximum absorbance at 512 nm against reagent blank, while method B is based on the oxidation of diacerein with potassium permanganate in an alkaline medium giving a pink-colored chromogen, which shows maximum absorption at 497.5 nm. Beer’s law was obeyed in the concentration range of 4 – 20 µg/ml for both methods A and B. Results of the analysis were validated statistically, and by recovery studies.

## INTRODUCTION

Diacerein is chemically 4, 5-diacetyloxy-9, 10-dioxo-anthracene-2-carboxylic acid, and is a disease modifying anti-rheumatoid drug used in the treatment of Osteoarthritis and chronic inflammatory arthritis.[1] Many methods have been described in the literature for the determination of diacerein with other drugs, individually and in combination.[2–5] In the present study an attempt has been made to develop new colorimetric methods for the estimation of diacerein in capsule dosage forms. Method A is based on the reaction of diacerein with the Folin-Ciocalteu reagent (FC reagent), in the presence of 0.5 N sodium hydroxide solution, giving a pink-colored chromogen, which shows maximum absorbance at 512 nm against a reagent blank, Method B involves oxidation of diacerein with potassium permanganate in the presence of 0.1 N sodium hydroxide, also yielding a pink-colored chromogen, which shows maximum absorption at 497.5 nm against reagent blank.

## MATERIALS AND METHODS

An ELICO model SL 164 double beam UV/Vis Spectrophotometer, with a spectral width of 2 nm, wavelength accuracy of 0.5 nm, and a pair of 10 mm matched quartz cells, was used to measure the absorbance of the resulting solutions. Diacerein (Micro Laboratoriess, Hosur, India), Folin-Ciocalteu’s (FC) reagent, 0.5 N sodium hydroxide solution, 0.1 N sodium hydroxide solution, potassium permanganate, and double glass-distilled water, were used in the study. Solutions of potassium permanganate (0.05% w/v) and sodium hydroxide (0.1 N and 0.5 N) were freshly prepared in distilled water.

### Working standard of drug and sample solutions

The standard stock solution of diacerein was prepared by dissolving 10 mg of diacerein in a 100 ml volumetric flask, using 10 ml of dimethylsulfoxide (DMSO), and then the volume was made up to the mark with ethanol (100 µg/ml). In case of formulation, two brands of commercially available capsules were analyzed by the proposed methods. Ten capsules of diacerein each containing 50 mg of accurately weighed powder, equivalent to 50 mg of diacerein were taken.

## ASSAY

### Method A

To a series of 10 ml volumetric flasks, volumes of standard diacerein solution (100 µg/ml), ranging from 0.4 to 2 ml, were added. Next, 2 ml of FC reagent was added and kept for five minutes. The volume was made up to 10 ml with 0.5 N NaOH. The absorbance of the pink-colored species was measured at 512 nm against a reagent blank. A calibration curve was constructed. Similarly the absorbance of the sample solution was measured and the amount of diacerein was determined by referring to the calibration curve.

### Method B

Aliquots of 0.4 to 2 ml portions of standard solution were transferred to a series of 10 ml standard flasks. To each, 2 ml of 0.05% potassium permanganate solution was added and shaken for 20 minutes. Next, 1 ml of 0.1 N sodium hydroxide was added, shaken well, and the volume was adjusted to 10 ml with 0.1 N sodium hydroxide. The absorbance of the solution was measured at 497.5 nm against a reagent blank and calibration curve was plotted. Similarly, the absorbance of the sample solution was measured and the amount of diacerein was determined by referring to the calibration curve.

## RESULTS AND DISCUSSION

**Figure 1:**
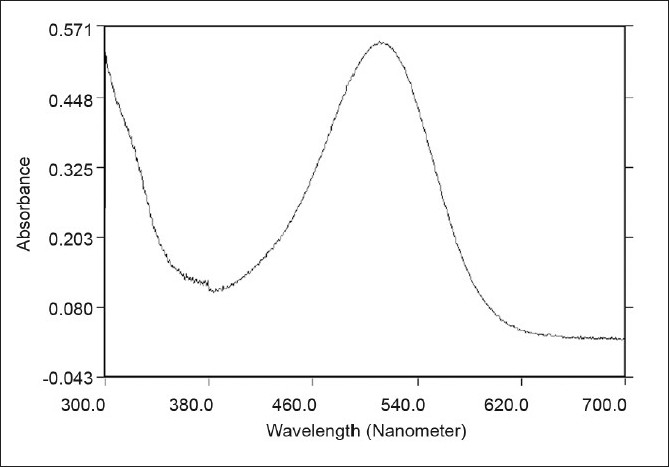
A typical spectra of diacerein by method A (20 µg/ml)

**Figure 2:**
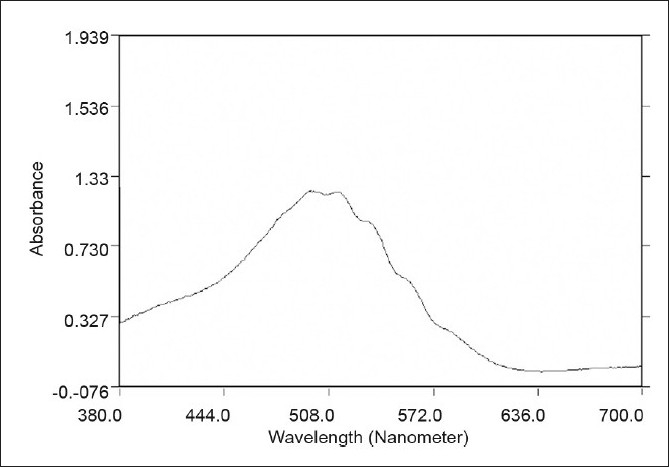
A typical spectra of diacerein by method B (10 µg/ml)

The optical characteristics such as absorption maxima, Beer’s law limits, Correlation coefficient (r), slope (m), y-intercept (c), molar absorptivity, and Sandell’s sensitivity, calculated from five replicate readings, for the proposed methods, are incorporated in Table 1. The molar absorptivity and Sandell’s sensitivity values show the sensitivity of both the methods. A typical spectrum of diacerein for both proposed methods A and B is given in Figures 1 and 2, respectively. The analysis results of the marketed formulations are in good agreement for both method A and method B [Table 2]. To test the accuracy and reproducibility of the proposed method, recovery experiments were carried out by adding known amounts of the drug to the preanalyzed formulation and reanalyzing the mixture by the proposed method. The results are shown in Table 2. A stability study of the chromogen was carried out by measuring the absorbance values at intervals of 10 minutes to six hours and it was found to be stable for four hours, for both the methods. The reproducibility, repeatability, and accuracy of these methods were found to be good, which was evident from the low percent relative standard deviation values (0.3327 for method A and 0.8432 for method B). The percentage recovery obtained (99.48 – 101.26 for method A and 98.43 – 99.22 for method B) indicated non-interference from the common excipients, including the lactose used in the formulations. Thus, the developed methods are simple, sensitive, accurate, and precise and can be successfully applied for the routine estimation of diacerein in pharmaceutical dosage forms.

**Table 1:** Validation parameters

Observation	Method A	Method B
Absorption maxima (nm)	512	497.5
Beer’fs law limit (µg/ml)	4 – 20	4 – 20
Correlation coefficient	0.9992	0.9994
Molar extinction coefficient (l/mol.cm)	2.0366 × 104	1.8893 × 104
Sandell’s selectivity (µg/cm^2^/0.001	0.0181	0.0195
Absorbance unit)		
Regression equation (y = mx + c)		
Slope (m)	0.053	0.047
Intercept (c)	0.047	0.072
Precision (% Relative Standard	0.3327	0.8432
Deviation)*		

* %relative standard deviation of five observations

**Table 2:** Evaluation of diacerein in pharmaceutical dosage forms

Sample	Labeled	Amount obtained (mg)*		% Recovery **	
		Method A	Method B	Method A	Method B
Brand A	50	49.32 ± 0.475	50.62 ± 0.833	99.48 ± 0.531	98.43 ± 0.947
Brand B	50	50.96 ± 0.652	49.79 ± 0.577	101.26 ± 0.732	99.22 ± 0.743

* Mean and % relative standard deviation of five observations,

** Mean and % relative standard deviation of five observations. (50 mg of diacerein was added and recovered)

## CONCLUSION

The present study shows that the methods are fast, sensitive, selective, precise, and do not require any expensive or sophisticated apparatus. It does not suffer from the interference of excipients. Hence, it can be used for a routine analysis of diacerein in bulk as well as in pharmaceutical preparations.
